# Explaining Within- vs Between-Population Variation in Child Anthropometry and Hemoglobin Measures in India: A Multilevel Analysis of the National Family Health Survey 2015–2016

**DOI:** 10.2188/jea.JE20190064

**Published:** 2020-11-05

**Authors:** Justin Rodgers, Rockli Kim, S. V. Subramanian

**Affiliations:** 1 Harvard Center for Population & Development Studies, Cambridge, MA, USA; 2 Division of Health Policy and Management, College of Health Sciences, Korea University, Seoul, Republic of Korea; 3 Department of Public Health Sciences, Graduate School, Korea University, Seoul, Republic of Korea; 4 Department of Social and Behavioral Sciences, Harvard T.H. Chan School of Public Health, Boston, MA, USA

**Keywords:** child undernutrition, child growth, anemia, multilevel modeling, variation, inequalities, India

## Abstract

**Background:**

The complex etiology of child growth failure and anemia—commonly used indicators of child undernutrition—involving proximate and distal risk factors at multiple levels is generally recognized. However, their independent and joint effects are often assessed with no clear conceptualization of inferential targets.

**Methods:**

We utilized hierarchical linear modeling and a nationally representative sample of 139,116 children aged 6–59 months from India (2015–2016) to estimate the extent to which a comprehensive set of 27 covariates explained the within- and between-population variation in height-for-age, weight-for-age, weight-for-height, and hemoglobin level.

**Results:**

Most of the variation in child anthropometry and hemoglobin measures was attributable to within-population differences (80–85%), whereas between-population differences (including communities, districts, and states) accounted for only 15–20%. The proximate and distal covariates explained 0.2–7.5% of within-population variation and 2.1–34.0% of between-population variation, depending on the indicator of interest. Substantial heterogeneity was observed in the magnitude of within-population variation, and the fraction explained, in child anthropometry and hemoglobin measures across the 36 states/union territories of India.

**Conclusions:**

Policies and interventions aimed at reducing between-population inequalities in child undernutrition may require a different set of components than those concerned with within-population inequalities. Both are needed to promote the health of the general population, as well as that of high-risk children.

## INTRODUCTION

Poor nutritional status in early childhood remains highly prevalent in low- and middle-income countries (LMICs)^1^^,^^2^ despite continued attention by global agencies, as exemplified by the Millennium Development Goals,^3^ Global Nutrition Targets 2025,^4^ and Sustainable Development Goals,^5^ as well as commitments from national governments in countries like India.^6^ Child growth failures and anemia are the most commonly used indicators of nutritional status in children, especially in low- and middle-income countries.^7^ Child undernutrition—as measured by child anthropometry and hemoglobin level—result from a series of complex interactions between socioeconomic conditions, inadequate dietary intake of key nutrients, and exposure to infectious diseases^1^^,^^8^^–^^11^ and are known to be detrimental to long-term health, human capital potentials, and economic progress for individuals and societies.^12^ Existing frameworks on causes of child malnutrition generally recognize the complex etiology of suboptimum growth with proximate and distal risk factors operating at multiple levels.^1^^,^^13^ Yet, the independent and joint effects of such risk factors on child undernutrition are often assessed with no clear conceptualization of the inferential targets.^14^

Applying individual and population perspectives in tandem to understand within- and between-population variations in health is potentially important in three aspects. First, from a theoretical perspective, the determinants of population averages may be fundamentally different from the determinants of individual cases.^15^^–^^17^ Despite the inter-relatedness of these two types of inferential questions, a population-based perspective to health dominates the epidemiologic literature and results in an exclusive focus on the comparison of mean values of health outcomes and exposures between populations,^16^^,^^18^ often defined as countries in global health research.^14^ At the same time, the within-population distribution is assumed be constant over time and across different populations, so it is often overlooked as being uninformative.^15^^,^^19^

Second, in the few studies that have attempted to quantify and explain within- and between-population variance in adult and child anthropometry across LMICs, the majority of variation (80–90%) was found to be attributed to within-population differences as opposed to between-population differences.^17^^,^^20^ Despite the disproportionately large within-population variation, only 2% was explained by basic socioeconomic factors for women’s body mass index (BMI) across 58 LMICs,^17^ and, similarly, only 1% was explained by mean values of maternal covariates of child anthropometric status and failure across 57 LMICs.^20^ Moreover, the within-population variability and its systematic components were found to be heterogeneous across countries in both studies.^17^^,^^20^

Third, a sizeable magnitude of the within-population differences may be systematically patterned^21^ and increase over time,^22^ raising further concerns for clearly defined targets of inference for policies and interventions.^14^ For instance, interventions aimed to reduce between-population inequalities may require an entirely different set of components than those aimed to reduce within-population inequalities, and both are needed to promote the health of the general population, as well as that of high-risk individuals.^20^

The present study aims to build on this nascent area of research by exploring the within- and between-population variation in child undernutrition, as measured via anthropometric status and hemoglobin level. We specifically advance prior work^20^ by utilizing the latest nationally representative survey from India (2015–2016) and considering a more comprehensive set of 27 socioeconomic and maternal/paternal covariates to estimate their total and individual contributions to explain the within- and between-population differences in child anthropometry and hemoglobin measures across all India and by 36 states and union territories.

## METHODS

### Survey data and study population

**Figure 1.**
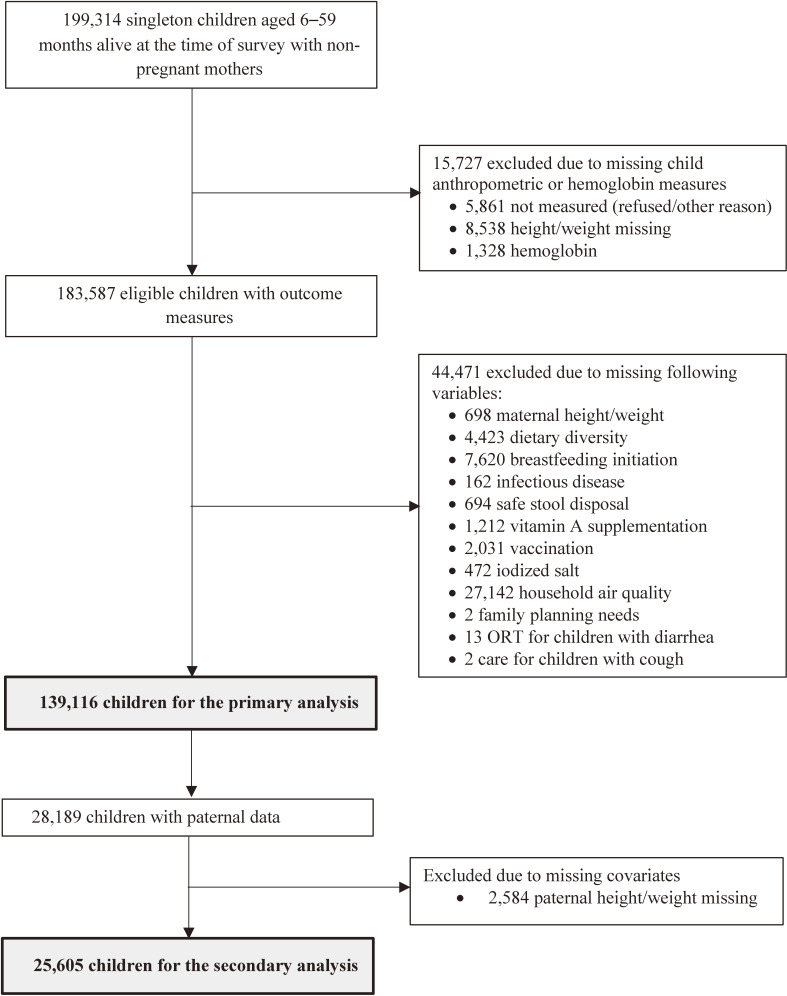
Flow diagram showing exclusions and final sample sizes of the study population, Indian National Family Health Survey 2015–2016

Data for this study were derived from the 4^th^ National Family Health Survey (NFHS-4), also equivalent to the 2015–2016 Demographic Health Survey (DHS) for India. The NFHS-4 was implemented with support from the Government of India Ministry of Health and Family Welfare and collected data related to child/maternal health, fertility, health-related behaviors and attitudes, household and environmental characteristics, nutrition, gender, women’s empowerment, and domestic violence.^23^ The NFHS-4, for the first time, included data from all 640 districts and 36 states and union territories within the country.^23^ Survey respondents were selected following a stratified two-stage sampling frame by states and urban and rural areas within each state.^24^ Out of a total of 199,314 children aged 6–59 months eligible for the study, 14,399 children were excluded for missing height or weight measures, 1,328 were excluded for missing hemoglobin data, and 44,471 children were removed due to missing information on one or more of the selected covariates, yielding a final analytic sample of 139,116 children. A subset of 25,603 children with additional information on paternal data was used for a secondary analysis (Figure 1).

### Defining within- and between-population units of inference

In this study, the within-population (between-individual) unit of inference refers to children. The between-population unit of inference collectively refers to communities, districts, and states given the hierarchical nature of the NFHS-4 data, as well their administrative, geographic, and political significance. By survey design, children in our final analytic sample were hierarchically nested within 26,986 communities across 640 districts and 36 states/union territories. In India, communities (or the primary sampling units) represent the local environment of villages for rural areas and urban frame survey blocks for urban areas.^23^ Districts are the lowest administrative unit at which the elected district councils plan the provision of diverse services and infrastructures, and states are the political unit at which federal policies operate.^25^ The selected units in our analysis, therefore, are consistent with prior multilevel analysis of India,^25^ and our conceptualization of within- and between-population units of inference aligns with recent studies.^17^^,^^20^

### Outcome variables

The primary outcome variables for this study were child anthropometry and hemoglobin measures, which are routinely used as proxy indicators of child undernutrition in the literature. Child anthropometry measures based on height and weight are important indicators for assessing population level nutrition status and diagnosing individual children who are undernourished, as well as for designing and evaluating programs and policies.^7^^,^^26^ Hemoglobin, a common marker for anemia, may represent underlying poor nutrition, vitamin and mineral (especially iron) deficiencies, inflammation, or disease.^11^ In the NFHS-4, height, weight, and hemoglobin level were objectively measured by field interview teams. Weight was measured using digital solar-powered scales along with adjustable Shorr measuring boards.^23^ Standing height was obtained for children older than 24 months while recumbent length was measured with children lying on the board placed on a flat surface for children younger than 24 months.^23^ The raw height and weight measures were transformed into age- and sex-specific z-scores using the WHO child growth standards^26^: height-for-age z-scores (HAZ), weight-for-age z-scores (WAZ), and weight-for-height z-scores (WHZ). Measures of hemoglobin were also standardized (HZ) using the z-score method. For an additional analysis, the dichotomous form of each measure was constructed: stunting defined as HAZ < −2 standard deviation (SD); underweight as WAZ < −2SD; wasting as WHZ < −2SD; and anemia as the original hemoglobin level <11 g/dL, according to the WHO criteria for children aged 6–59 months.^1^^,^^27^

### Independent variables

To assess the maximum upper bound of the systematic components in variability of child anthropometry and hemoglobin level, a total of 24 covariates related to child, maternal, and household characteristics were selected for the primary analysis based on conceptual frameworks on proximate and distal determinants of child undernutrition,^1^^,^^13^ prior epidemiological studies,^8^^,^^9^^,^^28^^–^^33^ and components of nutrition interventions and programs.^10^^,^^34^^–^^36^ Three additional paternal characteristics were included for the secondary analysis, hence a total of 27 covariates. All variables are summarized in Table 1.

**Table 1.** List of covariates included in the primary and secondary analysis

**Primary analysis: 24 covariates**	

• Child’s Age	Categorized as 6–11, 12–23, 24–35, 36–47, and 48–59 months.

• Child’s Sex	A binary variable for boys and girls.

• Child’s birth order	Categorized as 1^st^, 2^nd^ or 3^rd^, 4^th^ or 5^th^, and 6^th^ or above.

• Place of residence	Census based urban versus rural.

• Household wealth index	In the NFHS-4, household wealth index was created using principal component analyses of household characteristics and assets, and categorized into quintiles.

• Maternal education	Categorized in five levels: no schooling, primary, secondary, higher secondary, and college education.

• Maternal height	Women’s height was obtained directly by field interview teams using adjustable Shorr measuring boards, and was categorized as: <145, 145–149.9, 150–154.9, 155–159.9, and ≥160 cm.

• Maternal BMI	Women’s weight was measured using digital Secascales, and maternal BMI was categorized as <18.5, 18.5–24.9, and ≥25 kg/m^2^.

• Maternal age at marriage	Defined dichotomously for married or cohabitating mothers using the age of 18 years as cutoff.

• Dietary diversity	Based on a 24-hour recall of food intake in the NFHS questionnaire, a score for child’s dietary diversity was developed by assigning 1 point for consumption of milk, meat, lentils, starchy staples, vitamin A fruits, other fruits, dairy, and oils/fats/butter, and the score was grouped into quintiles.

• Timing of breastfeeding initiation	A dichotomous variable for initiating breastfeeding ≥1 hour of birth or <1 hour of birth.

• Source of drinking water	A dichotomous variable indicating safe source of drinking water for water piped into dwelling or yard/plot, public tap/standpipe, tube well or borehole, protected well or spring, rain water, and bottled water, and unsafe otherwise.

• Sanitation facility	A dichotomous variable indicating improved sanitation facility for households with access to flush to piped sewer system, septic tank, or pit latrine, ventilated improved pit latrine, pit latrine with slab, and composting toilet, and unimproved otherwise.

• Stool disposal	A dichotomous variable indicating safe or unsafe disposal of child’s stools.

• Infectious disease	A dichotomous variable indicating whether the child experienced infectious disease (eg, diarrhea, cough/fever) two weeks prior to the survey.

• Household air quality	A categorical variable indicating higher air quality for households using non-solid fuels, lower air quality for using solid fuels in separate kitchen, and the worst quality for using solid fuels in non-separate kitchen.

• Use of iodized salt	A dichotomous variable indicating whether the household used iodized salt.

• Vitamin A supplementation	A dichotomous variable indicating whether vitamin A supplementation was given to the child.

• Full vaccination	A dichotomous variable indicating whether the child was fully vaccinated with measles, BCG, DPT 3, and Polio 3.

• Family planning needs	Unmet need for family planning was coded as 1 if woman reported unmet need for spacing or limiting, and 0 otherwise.

• Skilled birth attendant	Indicator variable was created for births attended by skilled health personnel (doctor, nurse, or midwife).

• Antenatal care (ANC) visits	The number of ANC visits was categorized as <4 or ≥4 based on the new WHO recommendation.

• Oral rehydration therapy (ORT) for diarrhea	A binary variable indicating whether ORT was given for a child with diarrhea.

• Care seeking for cough/fever	A binary variable indicating whether care was sought for a child with cough as a proxy measure for care seeking for pneumonia.

**Secondary analysis: 3 additional covariates**	

• Paternal education	Categorized in five levels: no schooling, primary, secondary, higher secondary, and college education.

• Paternal height	Men’s height was obtained directly by field interview teams using adjustable Shorr measuring boards, and was categorized as: <155, 155–159.9, 160–164.9, 165–169.0, and ≥170 cm.

• Paternal BMI	Men’s weight was measured using digital Secascales, and paternal BMI was categorized as <18.5, 18.5–24.9, and ≥25 kg/m^2^.

### Statistical analysis

We utilized multilevel statistical models to simultaneously assess factors driving the between- and within-population differences in child anthropometric status and hemoglobin measures, while also accounting for the complex sampling design of the data.^37^ This statistical approach has been widely used in public health literature where the scientific interest is in simultaneously examining compositional and contextual factors on health outcomes.^38^^–^^41^ Its application has been recently extended to better understand between- and within-population differences in adult women’s BMI^17^ and child anthropometric failures.^20^

To fully account for the hierarchical structure of the data, and being informed of important administrative and geographic units in India from prior literature, we specified the following random effects linear regression model with child *i* (level-1) nested within community *j* (level-2), district *k* (level-3), and state *l* (level-4):

$$Y_{ijkl}=\beta_{0}+\beta {\text{X}}_{ijkl}+(e_{0ijkl}+u_{0jkl}+v_{0kl}+f_{0l})$$

where *Y* represents the outcome (HAZ, WAZ, WHZ or HZ); *X* is a vector of covariates; and *e*_0_*_ijkl_*, *u*_0_*_jkl_*, *v*_0_*_kl_*, and *f*_0_*_l_* are residuals specific to each level (individual, community, district, and state) respectively. Under the independently and identically distributed (iid) assumption, each set of residuals follows a normal distribution with a mean of 0 and a variance of $e_{0ijkl}∼N(0,\sigma_{e_{0}}^{2})$, $u_{0jkl}∼N(0,\sigma_{u_{0}}^{2})$, $v_{0kl}∼N(0,\sigma_{v_{0}}^{2})$, and $f_{0l}∼N(0,\sigma_{f_{0}}^{2})$. The variance estimates for communities, districts, and states were summed for the between-population variation (ie, $\sigma_{u_{0}}^{2}+\sigma_{v_{0}}^{2}+\sigma_{f_{0}}^{2}$). Hence, the proportion of variation in the outcome attributable to the between-population differences was calculated as $\left(\frac{\sigma_{u_{0}}^{2}+\sigma_{v_{0}}^{2}+\sigma_{f_{0}}^{2}}{\sigma_{e_{0}}^{2}+\sigma_{u_{0}}^{2}+\sigma_{v_{0}}^{2}+\sigma_{f_{0}}^{2}}\right)\times 100$ and the proportion attributed to the within-population differences as $\left(\frac{\sigma_{e_{0}}^{2}}{\sigma_{e_{0}}^{2}+\sigma_{u_{0}}^{2}+\sigma_{v_{0}}^{2}+\sigma_{f_{0}}^{2}}\right)\times 100$. Variance partitioning coefficient, or intraclass correlation coefficient, is often the parameter of interest in conventional multilevel analysis that is interpreted for the significance of variability across different units of inference.^42^^,^^43^

For the primary analysis, we first adjusted for child’s age- and sex-only (model 1), and then additionally included all 22 covariates together in a combined model (model 2). The difference in the within- and between-population variation from model 1 to model 2 was compared using a percent-change calculation, in order to quantify the contribution of covariates in explaining variation in child anthropometry and hemoglobin. In multilevel analysis, changes in the value of between-group variation after adding individual-level variables are examined to understand the degree to which group variability is accounted for by omitted individual-level variables.^42^^,^^43^ We applied this assessment to calculate percent explained as the change in variance estimates from model 1 to 2: for example, $\left(\frac{[\sigma_{{\text{u}}_{0}}^{2}+\sigma_{{\text{v}}_{0}}^{2}+\sigma_{{\text{f}}_{0}}^{2}{]}_{\text{model }1}-[\sigma_{{\text{u}}_{0}}^{2}+\sigma_{{\text{v}}_{0}}^{2}+\sigma_{{\text{f}}_{0}}^{2}{]}_{\text{model }2}}{[\sigma_{{\text{u}}_{0}}^{2}+\sigma_{{\text{v}}_{0}}^{2}+\sigma_{{\text{f}}_{0}}^{2}{]}_{\text{model }1}}\right)\times 100$ for percent explained in between-population variation and $\left(\frac{[\sigma_{{\text{e}}_{0}}^{2}{]}_{\text{model }1}-[\sigma_{{\text{e}}_{0}}^{2}{]}_{\text{model }2}}{[\sigma_{{\text{e}}_{0}}^{2}{]}_{\text{model }1}}\right)\times 100$ for percent explained in within-population variation. We utilized cluster bootstrapping with 1,000 replicates to calculate 95% confidence intervals (CIs) for the percent-explained estimates. This approach takes random samples at each level of the data (ie, states, districts, communities, and individuals) in order to account for its hierarchical nature and the original sampling procedure.^44^^–^^46^ Cluster bootstrapping has been shown to yield consistent estimates of model parameters and variance components in multilevel models, as well as more closely mimic the variation properties of hierarchical data compared to other bootstrapping approaches.^45^^–^^47^

Additionally, state-specific analysis was conducted to assess the differential magnitude of variance explained by the same covariates across states. For the secondary analysis, we considered three additional paternal covariates (hence a total of 27 covariates) for a subset of the sample surveyed for father’s data (model 3). We also conducted two sensitivity analyses. First, each of the selected covariates was individually added to model 1 in a stepwise manner to assess which individual covariates explained the most between- and within-population differences. Second, multilevel logistic regression models were conducted to examine the binary outcome variables (stunting, wasting, underweight, and anemia). All analyses were conducted using the R programming. The cluster bootstrapping procedure described above was operationalized via a user-defined function in base R. All multilevel models were fit using the ‘lmer’ function from the ‘lme4’ package for fitting linear mixed effects models.^48^

### Ethics statement

The study was reviewed by Harvard T.H. Chan School of Public Health Institutional Review Board and was considered exempt from full review because the study was based on an anonymous public use data set with no identifiable information on the study participants.

## RESULTS

### Sample characteristics

Of 139,116 children included in the primary analysis, the overall mean values of HAZ, WAZ, WHZ, and HZ were −1.51 (SD, 1.63), −1.52 (SD, 1.21), −0.95 (SD, 1.35), and 0 (SD, 1), respectively. For the corresponding binary variables, 38.6% were stunted, 19.6% experienced wasting, 34.2% were underweight, and 55.2% were anemic.

### Variance decomposition

The total variance estimated from the age- and sex-adjusted model was 2.59 for HAZ, 1.44 for WAZ, 1.83 for WHZ, and 1.04 for HZ. Of the total variance in HAZ, 85.4% was attributable to the within-population differences while the remaining 14.6% was attributed to the between-population differences (Table 2). Similarly, 81.1% and 84.3% of the total variation in WAZ and WHZ was attributed to the within-population differences while 18.9% and 15.7% was attributed to the between-population differences, respectively. For HZ, 80.1% of the total variation was accounted for by within-population differences, and the remaining 19.9% was attributable to the between-population differences. The proportion of between-population variation broken down by states, districts, and communities is presented in eTable 1. In general, communities were more variable than states and districts for all outcomes.

**Table 2.** Variance estimates in child anthropometry and hemoglobin level using four-level random intercepts models, and % explained by a comprehensive set of covariates, National Family Health Survey (NFHS-4)

	**Primary Analysis (*n* = 139,116)**			**Secondary Analysis (*n* = 25,605)**		
	
	Variance estimates (95% CI)		% Explained (95% CI)	Variance estimates (95% CI)		% Explained (95% CI)
			
	Model 1	Model 2	Model 1 vs Model 2	Model 1	Model 3	Model 1 vs Model 3
**Height-for-age z-scores**						
Between-population	0.38 (0.37, 0.38)	0.25 (0.24, 0.26)	33.3 (32.6, 34.0)%	0.78 (0.76, 0.80)	0.63 (0.62, 0.64)	20.1 (18.7, 21.6)%
Within-population	2.21 (2.20, 2.22)	2.09 (2.07, 2.11)	5.4 (4.8, 6.1)%	1.86 (1.84, 1.88)	1.76 (1.74, 1.78)	5.2 (4.3, 6.1)%
**Weight-for-age z-scores**						
Between-population	0.27 (0.27, 0.27)	0.16 (0.15, 0.17)	39.4 (38.2, 39.6)%	0.49 (0.48, 0.51)	0.35 (0.34, 0.36)	28.9 (27.0, 30.7)%
Within-population	1.17 (1.16, 1.18)	1.08 (1.07, 1.09)	7.5 (7.3, 7.7)%	0.97 (0.96, 0.98)	0.90 (0.89, 0.91)	7.9 (6.7, 9.1)%
**Weight-for-height z-scores**						
Between-population	0.29 (0.29, 0.29)	0.25 (0.25, 0.25)	12.8 (12.1, 13.5)%	0.59 (0.58, 0.60)	0.53 (0.52, 0.54)	9.9 (9.2, 10.7)%
Within-population	1.55 (1.54, 1.56)	1.52 (1.51, 1.53)	2.0 (1.4, 2.6)%	1.27 (1.26, 1.28)	1.24 (1.23, 1.25)	2.4 (1.9, 2.9)%
**Standardized Hemoglobin**						
Between-population	0.21 (0.20, 0.22)	0.20 (0.19, 0.21)	2.1 (1.5, 2.8)%	0.46 (0.45, 0.47)	0.45 (0.44, 0.46)	1.6 (1.1, 2.0)%
Within-population	0.83 (0.83, 0.83)	0.83 (0.83, 0.83)	0.2 (0.0, 0.4)%	0.61 (0.60, 0.62)	0.61 (0.60, 0.62)	0.5 (0.2, 0.7)%

CI, confidence interval.

Model 1: age and sex.

Model 2: Model 1 + birth order, residence (urban/rural), household wealth, mother’s height, mother’s BMI, mother’s age at marriage, child’s dietary diversity, breastfeeding, drinking water availability, sanitation, safe stool disposal, infectious disease, household air quality, iodized salt, vitamin A supplementation, full vaccination, family planning, skilled birth attendant, antenatal care visits, child diarrhea in past 2 weeks, mother sought care for child with cough/fever.

Model 3: Model 2 + father’s height, father’s BMI, father’s education, father’s age.

% Explained refers to the change in variance estimates from Model 1 to Model 2 (or Model 3). For example, % explained in between-population variation from Model 1 and Model 2 is calculated $\left(\frac{[\sigma_{{\text{u}}_{0}}^{2}+\sigma_{{\text{v}}_{0}}^{2}+\sigma_{{\text{f}}_{0}}^{2}{]}_{\text{model }1}-[\sigma_{{\text{u}}_{0}}^{2}+\sigma_{{\text{v}}_{0}}^{2}+\sigma_{{\text{f}}_{0}}^{2}{]}_{\text{model }2}}{[\sigma_{{\text{u}}_{0}}^{2}+\sigma_{{\text{v}}_{0}}^{2}+\sigma_{{\text{f}}_{0}}^{2}{]}_{\text{model }1}}\right)\times 100$ and % explained in within-population variation from Model 1 and Model 2 is calculated as $(\frac{[\sigma_{{\text{e}}_{0}}^{2}{]}_{\text{model }1}-[\sigma_{{\text{e}}_{0}}^{2}{]}_{\text{model }2}}{[\sigma_{{\text{e}}_{0}}^{2}{]}_{\text{model }1}})\times 100$. 95% confidence intervals for % explained calculated via bootstrapping with 1,000 bootstrap re-samples.

### Variance explained by all covariates

Despite the large variation in HAZ observed within-populations, only 5.4% (95% CI, 4.8–6.1%) was explained by the addition of 22 sociodemographic and maternal health covariates (Table 2; the regression coefficients are presented in eTable 2). On the other hand, despite between-population variation accounting for a smaller proportion of the total variance, the addition of all covariates explained 33.3% (95% CI, 32.6–34.0%) of the between-population differences in HAZ. Similarly, adjusting for all covariates explained 7.5% (95% CI, 7.3–7.7%) of the within-population variation and 39.4% (95% CI, 38.2–39.6%) of the between-population variation in WAZ. A smaller fraction of the variation in WHZ was explained by the same set of covariates: 2.0% (95% CI, 1.4–2.6%) of within-population and 12.8% (95% CI, 12.1–13.5%) of between-population. For the standardized hemoglobin measure, the addition of sociodemographic and maternal covariates explained only 0.2% (95% CI, 0.0–0.4%) of the within-population variation and 2.1% (95% CI, 1.5–2.8%) of the between-population variation. A secondary analysis with a subset of 25,603 children showed that additional data on father’s height, BMI and education did not offer further explanation of the between- and within-population variation in all indicators of child undernutrition (Table 2; eTable 3).

### State-specific analysis

**Figure 2.**
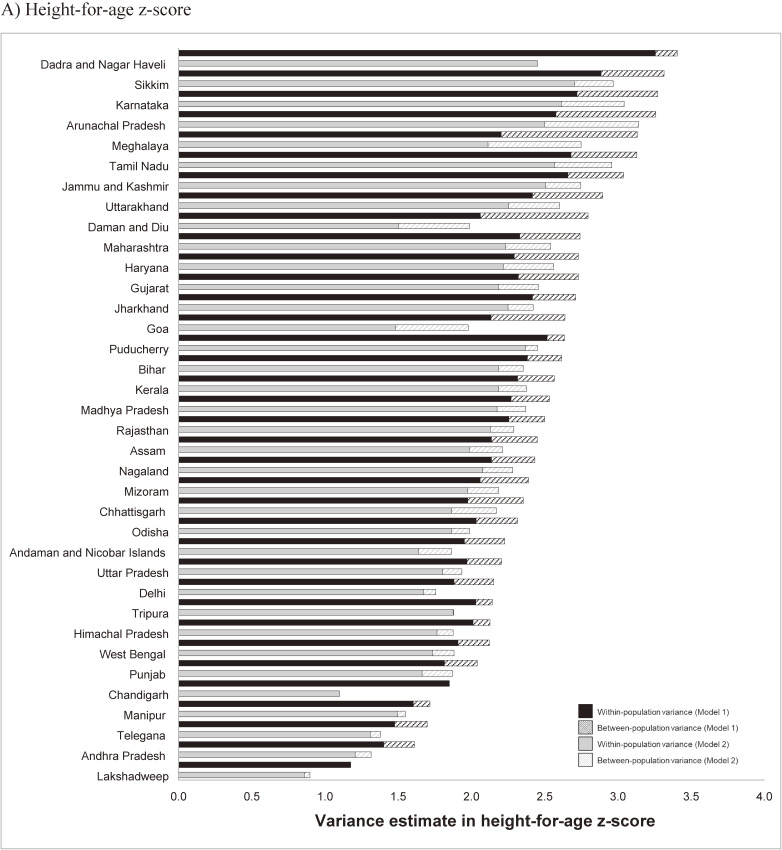

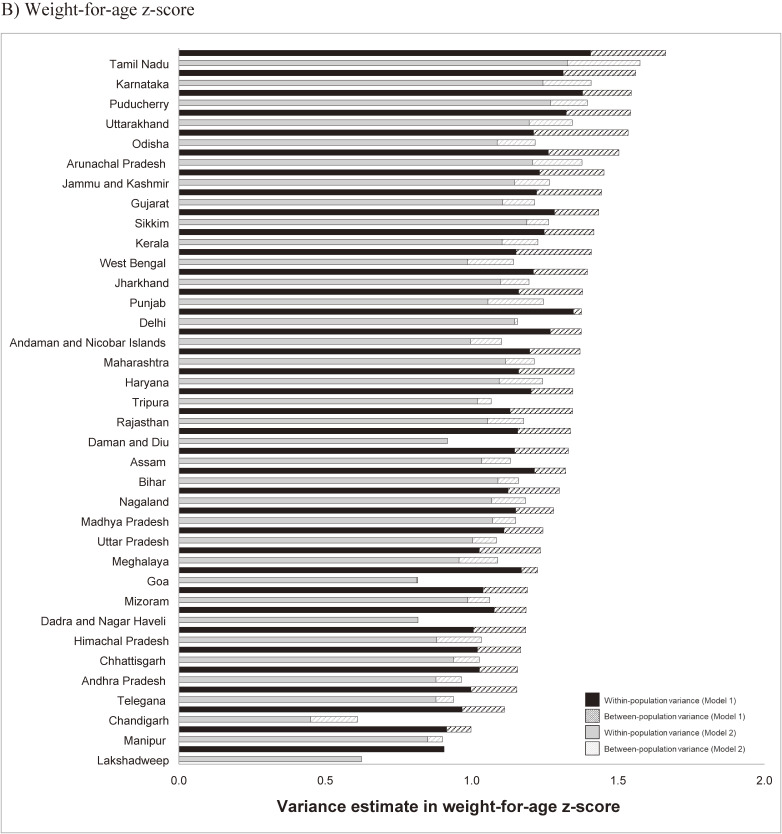

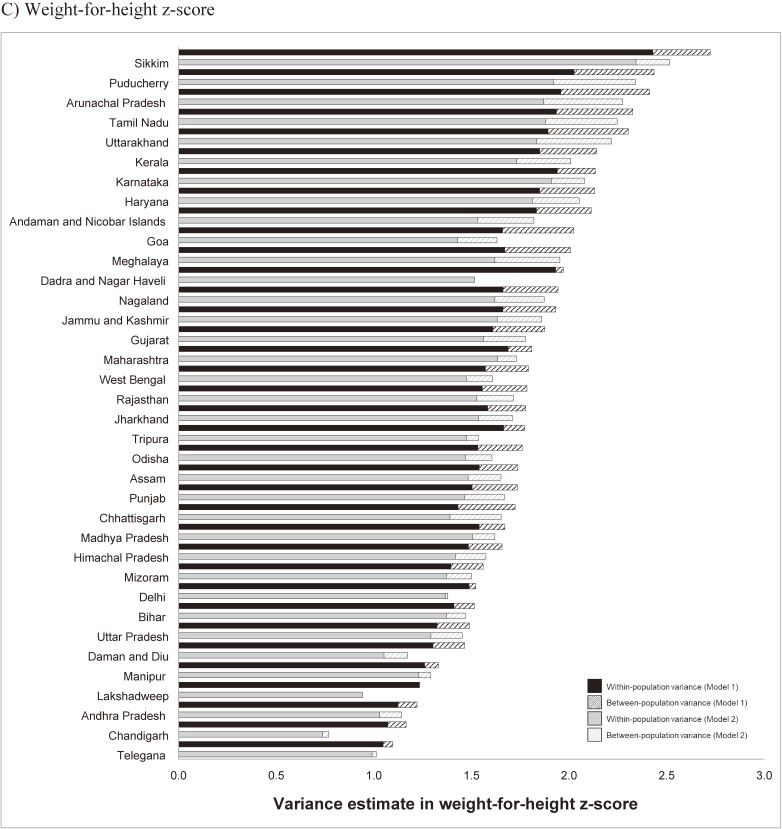

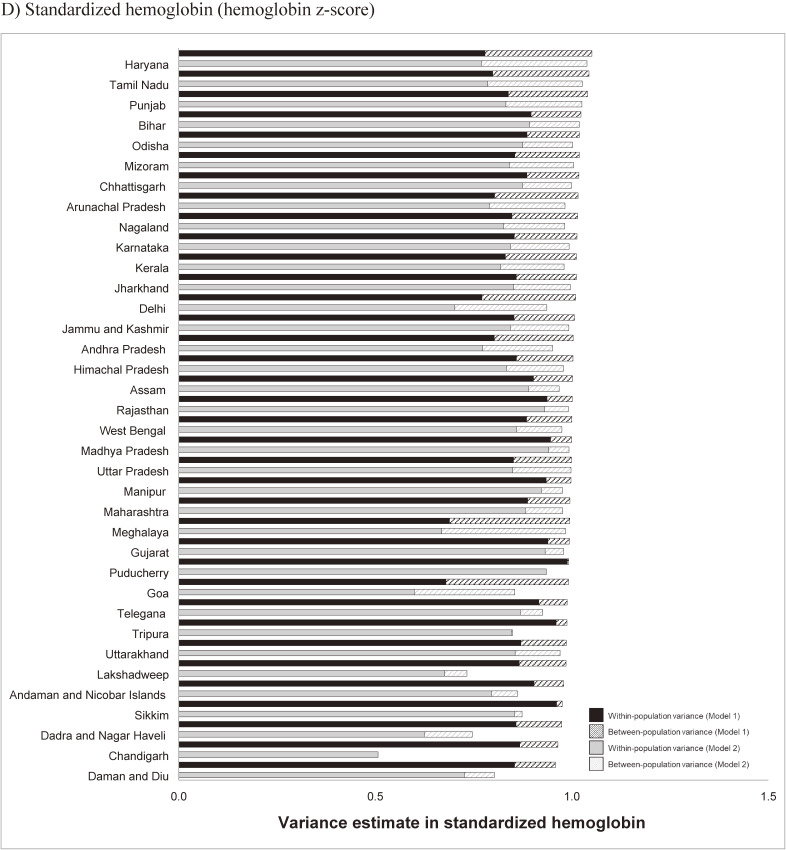
Between- and within-population variation in A) height-for-age z-score, B) weight-for-age z-score, C) weight-for-height z-score, and D) standardized hemoglobin from four-level random intercepts models before and after adjusting for a comprehensive set of covariates, National Family Health Survey (NFHS-4) Model 1: age and sex. Model 2: Model 1 + birth order, residence (urban/rural), household wealth, mother’s height, mother’s BMI, mother’s age at marriage, child’s dietary diversity, breastfeeding, drinking water availability, sanitation, safe stool disposal, infectious disease, household air quality, iodized salt, vitamin A supplementation, full vaccination, family planning, skilled birth attendant, antenatal care visits, child diarrhea in past 2 weeks, mother sought care for child with cough/fever.

In state-specific analyses, we consistently found the majority of variation to be within-population, which was poorly explained by the comprehensive set of covariates. The within-population variance in HAZ from age- and sex-adjusted models ranged from 1.18 in Lakshadweep to 3.26 in Dadra and Nagar Haveli, accounting for 70% of total variation in Meghalaya to close to 100% in Chandigarh (Figure 2A). The proportion of within-population variation in HAZ explained by covariate adjustment ranged from as low as 2.8% in Nagaland to more than 10% in 10 states, including up to 30.6% in Goa and 40.5% in Chandigarh. For the majority of states, 20% to 70% of the between-population variation in HAZ was explained by the same covariates. Likewise, the degree of variation and contribution of systematic components also varied substantially across states for WAZ (Figure 2B), WHZ (Figure 2C), and HZ (Figure 2D). The proportion of within-population variation in WAZ ranged from 79% in Odisha to 100% in Lakshadweep, of which <10% was explained by the covariates in 21 states. Despite the smaller between-population variation in WAZ across all states, >30% was explained in 26 states (Figure 2B). For WHZ, <5% of the within-population variation was explained across 23 states while >20% of the between-population variation was explained within 12 states (Figure 2C). The largest within-population and between-population variation in HZ was found in Puducherry (99.7%) and Goa (31.4%), respectively. The proportion explained by covariates ranged from <1% in 8 states, including Uttar Pradesh and Bihar, to 41.6% in Chandigarh for the within-population variation and from <1% in 8 states, including Sikkim and Dadra and Nagar Haveli, to almost 100% in Puducherry and Chandigarh for the between-population variation in HZ (Figure 2D).

### Additional analyses

Examining each covariate separately revealed differential ability to explain individual and population variance in child anthropometry and hemoglobin level. For HAZ, the percent explained in the between-population variance was greatest for the following covariates: household wealth (22.1%; 95% CI, 21.4–23.1%), maternal education (19.9%; 95% CI, 19.1–20.6%), household air quality (17.0%; 95% CI, 16.4–17.6%), maternal height (13.5%; 95% CI, 12.8–14.0%), sanitation (13.5%; 95% CI, 13.2–13.8%), safe stool disposal (11.0%; 95% CI, 10.5–11.6%), maternal BMI (9.7%; 95% CI, 9.3–10.1%), ANC visits (6.1%; 95% CI, 5.9–6.4%), urban/rural residence (5.7%; 95% CI, 5.4–6.1%), birth order (4.9%; 95% CI, 4.9–5.0%), skilled birth attendant (4.2%; 95% CI, 4.0–4.4%), and mother’s age at marriage (4.0%; 95% CI, 3.7–4.3%) (Table 3). The remaining covariates each explained <1% of the total between-population variance in HAZ. At the same time, all the covariates, except for maternal height (3.5%; 95% CI, 3.3–3.8%), household wealth (1.7%; 95% CI, 1.6–1.8%), and maternal education (1.2%; 95% CI, 1.1–1.4%), each explained <1% of the within-population variance in HAZ. Similar patterns were found for WAZ, WHZ, and HZ (Table 3).

**Table 3.** Contribution of individual covariates in explaining between- and within-population variation in child anthropometry and hemoglobin level, National Family Health Survey (NFHS-4; *n* = 139,116)

Covariates	**Height-for-age z-scores**		**Weight-for-age z-scores**		**Weight-for-height z-scores**		**Standardized Hemoglobin**	
			
	% Explained (95% CI)		% Explained (95% CI)		% Explained (95% CI)		% Explained (95% CI)	
			
	Between-population	Within-population	Between-population	Within-population	Between-population	Within-population	Between-population	Within-population
Birth order	4.9 (4.9, 5.0)%	0.2 (0.1, 0.3)%	3.8 (3.7, 5.0)%	0.2 (0.1, 0.3)%	0.3 (0.2, 0.4)%	0.1 (0.0, 0.1)%	0.3 (0.1, 0.4)%	0.0 (0.0, 0.0)%
Residence	5.7 (5.4, 6.1)%	0.0 (0.0, 0.0)%	4.8 (4.5, 5.1)%	0.0 (0.0, 0.0)%	0.7 (0.5, 0.9)%	0.0 (0.0, 0.0)%	0.6 (−0.6, −0.4)%	0.0 (−0.0, 0.0)%
Household Wealth	22.1 (21.4, 23.1)%	1.7 (1.6, 1.8)%	23.6 (22.9, 24.3)%	1.7 (1.5, 2.0)%	5.3 (5.3, 6.2)%	0.5 (0.4, 0.6)%	−2.0 (−3.0, −2.0)%	0.1 (0.0, 0.1)%
Maternal Education	19.9 (19.1, 20.6)%	1.2 (1.1, 1.4)%	19.6 (18.9, 20.3)%	1.2 (1.1, 1.3)%	3.8 (3.7, 4.2)%	0.3 (0.2, 0.5)%	3.4 (3.1, 3.7)%	0.1 (0.0, 0.2)%
Mother height	13.5 (12.8, 14.0)%	3.5 (3.3, 3.8)%	12.5 (12.0, 13.1)%	3.5 (3.2, 3.8)%	2.0 (1.9, 2.2)%	0.2 (0.1, 0.2)%	0.1 (0.1, 0.3)%	0.0 (0.0, 0.1)%
Mother’s BMI	9.7 (9.3, 10.1)%	0.5 (0.4, 0.5)%	19.7 (19.3, 20.2)%	0.5 (0.5, 0.6)%	10.8 (10.4, 11.4)%	1.2 (1.0, 1.4)%	1.0 (0.9, 1.2)%	0.0 (0.0, 0.1)%
Age at marriage	4.0 (3.7, 4.3)%	0.1 (0.1, 0.1)%	4.3 (4.1, 4.5)%	0.1 (0.0, 0.1)%	1.0 (0.9, 1.1)%	0.0 (0.0, 0.0)%	0.1 (0.1, 0.1)%	0.0 (−0.0, 0.1)%
Dietary diversity	0.1 (0.0, 0.1)%	0.0 (−0.1, 1.0)%	0.4 (0.3, 0.5)%	0.0 (0.0, 0.0)%	0.3 (0.2, 0.4)%	0.1 (0.0, 0.1)%	0.0 (0.0, 0.0)%	0.0 (−0.1, 0.1)%
Breastfeeding	0.0 (0.0, 0.1)%	0.5 (0.4, 0.6)%	−0.1 (−0.2, 0.0)%	0.5 (0.4, 0.6)%	−0.2 (−0.3, −0.1)%	0.0 (0.0, 0.0)%	0.0 (0.0, 0.1)%	0.0 (−0.1, 0.1)%
Drinking water	−0.1 (−0.2, −0.1)%	0.0 (0.0, 0.0)%	−0.2 (−0.2, −0.1)%	0.0 (0.0, 0.0)%	0.0 (−0.1, 0.0)%	0.0 (0.0, 0.0)%	0.0 (0.0, 0.0)%	0.0 (0.0, 0.0)%
Sanitation	13.5 (13.2, 13.8)%	0.0 (0.0, 0.0)%	17.8 (17.1, 18.5)%	0.0 (0.0, 0.0)%	5.5 (5.2, 6.0)%	0.1 (0.0, 0.1)%	2.3 (2.2, 2.5)%	0.0 (0.0, 0.0)%
Stool disposal	11.0 (10.5, 11.6)%	0.3 (0.2, 0.3)%	13.0 (12.3, 13.8)%	0.3 (0.3, 0.4)%	3.4 (3.2, 3.7)%	0.1 (0.0, 0.1)%	0.8 (0.7, 1.0)%	0.0 (0.0, 0.1)%
Infectious Disease	0.2 (0.0, 0.0)%	0.0 (0.0, 0.0)%	−0.1 (−0.2, −0.1)%	0.0 (−0.1, 0.1)%	−0.3 (−0.4, −0.2)%	0.1 (0.0, 0.1)%	0.0 (0.0, 0.1)%	0.0 (−0.0, 0.0)%
Home air quality	17.0 (16.4, 17.6)%	0.5 (0.4, 0.6)%	17.4 (17.0, 17.9)%	0.5 (0.4, 0.6)%	3.8 (3.6, 4.1)%	0.2 (0.1, 0.3)%	−0.3 (−0.6, −0.1)%	0.0 (0.0, 0.0)%
Iodized salt	0.5 (0.4, 0.6)%	0.0 (0.0, 0.0)%	0.8 (0.8, 0.9)%	0.0 (0.0, 0.0)%	0.3 (0.2, 0.4)%	0.0 (0.0, 0.0)%	0.2 (0.0, 0.3)%	0.0 (0.0, 0.0)%
Vitamin A suppl.	0.3 (0.3, 0.4)%	0.0 (0.0, 0.0)%	0.2 (0.2, 0.2)%	0.0 (0.0, 0.0)%	−0.1 (−0.1, −0.1)%	0.0 (0.0, 0.0)%	0.0 (−0.0, 0.0)%	0.0 (0.0, 0.0)%
Full vaccination	0.3 (0.2, 0.3)%	0.0 (0.0, 0.0)%	0.4 (0.4, 0.5)%	0.0 (0.0, 0.0)%	0.0 (0.0, 0.1)%	0.0 (0.0, 0.0)%	0.2 (0.2, 0.3)%	0.0 (0.0, 0.0)%
Family planning	0.0 (0.0, 0.0)%	0.0 (0.0, 0.0)%	0.0 (0.0, 0.1)%	0.0 (0.0, 0.0)%	−0.0 (−0.1, 0.1)%	0.0 (0.0, 0.0)%	0.0 (−0.1, 0.0)%	0.0 (0.0, 0.0)%
Birth attendant	4.2 (4.0, 4.4)%	0.0 (0.0, 0.0)%	3.5 (3.3, 3.7)%	0.0 (0.0, 0.0)%	0.3 (0.2, 0.4)%	0.0 (0.0, 0.0)%	−0.2 (−0.3, −0.1)%	0.0 (0.0, 0.0)%
Antenatal care visits	6.1 (5.9, 6.4)%	0.1 (0.0, 0.0)%	5.4 (5.1, 5.7)%	0.1 (0.0, 0.0)%	0.2 (0.1, 0.3)%	0.2 (0.1, 0.3)%	0.0 (−0.1, 0.0)%	0.0 (0.0, 0.0)%
Child diarrhea	0.2 (0.2, 0.3)%	0.0 (0.0, 0.0)%	0.4 (0.3, 0.4)%	0.0 (0.0, 0.0)%	0.2 (0.1, 0.2)%	0.1 (0.0, 0.2)%	0.1 (0.1, 0.2)%	0.0 (0.0, 0.0)%
Child cough/fever	0.0 (0.0, 0.1)%	0.0 (0.0, 0.0)%	−0.1 (−0.1, 0.0)%	0.0 (0.0, 0.0)%	−1.5 (−0.2, −0.1)%	0.0 (0.0, 0.0)%	0.0 (0.0, 0.0)%	0.0 (0.0, 0.0)%

BMI, body mass index; CI, confidence interval.

Each value represents the percent reduction in response to the addition of the listed risk factor over and above adjustment for age and sex.

Between-population columns give the percent reduction for the total population-level (district+community+state). 95% confidence intervals for percent reductions calculated via bootstrapping with 1,000 bootstrap re-samples.

The variance partitioning by between- and within-population, as well as the contribution of the covariates, remained consistent in the multilevel logistic regression models for stunting, underweight, and wasting (eTable 4). Most of the variation (83–89%) was attributed to the within-population differences. Of this between-population variation, 4.1–62.2% was explained by the combined set of covariates.

## DISCUSSION

We present three salient findings from this comprehensive assessment of variation in child anthropometry and hemoglobin measures in India. First, most of the variation in HAZ, WAZ, WHZ, and HZ was attributable to within-population differences (80–85%), whereas the between-population differences (including communities, districts, and states) accounted for a much smaller portion of the total variance (15–20%). Second, a comprehensive set of proximate and distal covariates explained 0.2–7.5% of the within-population variation and 2.1–39.4% of the between-population variation, depending on the indicator of interest. Third, while a disproportionately large unexplained within-population variation in child anthropometry and hemoglobin measures was consistently found across all 36 states/union territories, both the magnitudes of variability as well as their systematic components were substantially heterogeneous.

Our analysis utilized the latest nationally representative data from India that are generally known to be of high quality.^23^ While the dataset was large and high quality, there were missing observations in the data, which we excluded from analysis. This may bias the estimates in our multilevel models if missing observations are not missing completely at random. Additionally, while anthropometry and hemoglobin measures were objectively measured by trained field investigators, most of the covariates were self-reported by mothers.^23^ Adjusting for self-reported indicators with potential measurement errors, likely being random, may lead to conservative estimates of the proportion explained in variation. The variance partitioning coefficient in any multilevel analysis is inherently sensitive to the choice of random effects simultaneously considered in the model.^49^ The choice of multilevel structure in our analysis was informed by both the hierarchical nature of the NFHS-4 data structure as well as a review of literature on the multiple units of administrative, geographic, and political significance in India.^25^ The extent to which variation in child anthropometry and hemoglobin is explained inevitably depends on the selected set of variables in the data set and their quality. Nevertheless, our findings largely align with a prior study on anthropometric growth failure across multiple LMICs.^20^ We found a larger fraction of the within-population variation in child undernutrition indicators being explained, given a more comprehensive set of covariates considered.

Our findings are highly relevant to the current policy discussion in India for the following reasons. Child anthropometric failures and anemia are among the key indicators used by the National Institution for Transforming India (NITI) to monitor progress in child nutrition.^6^ For instance, the National Nutrition Strategy (NNS) explicitly targets to prevent and reduce prevalence of underweight (ie, low weight-for-age) in children (0–3 years) by three percentage points per annum by 2022 and anemia among young children, adolescent girls, and women in the reproductive age group (15–49 years) by one-third of the current level by 2022.^6^ Improvement in the mean levels, as well as reduction in the variability, of anthropometric status and hemoglobin level is necessary to achieve these goals.^14^^,^^20^ That is, both universal strategies affecting the general well-being of the population and targeted strategies to specifically intervene on the high-risk subgroups should be conceptualized and practiced together.^14^^,^^15^^,^^18^^,^^20^^,^^36^

While we consistently found larger unexplained within-population differences across all indicators of child undernutrition, there were some notable differences supporting prior studies on differential causes and patterns of HAZ, WAZ, and WHZ.^8^^,^^9^^,^^30^ The within-population differences remained the greatest for HAZ, even after adjustment for a comprehensive set of covariates, followed by WHZ, WAZ, and HZ. The between-population differences from the mutually adjusted models were equivalent for HAZ and WHZ and smaller for HZ and WAZ. At the same time, a larger proportion of the between- and within-population variation was explained for WAZ and HAZ. The larger overall variability in HAZ may reflect the nature of chronicity in deprivation that are known to be more strongly correlated with maternal nutrition as well as socio-environmental conditions.^8^^,^^29^ Indeed, maternal nutritional status (BMI and height) and socioeconomic factors (household wealth, maternal education, safe stool disposal, and household air quality) explained a larger fraction of the between-population variance for HAZ and WAZ, but not for WHZ and HZ. Variability in WHZ perhaps could be better explained in the presence of data on infectious diseases and causes of acute starvations.^30^ We found the smallest variability in HZ given the universally high prevalence of anemia across all India.^50^^,^^51^ Multifarious etiology of anemia, ranging from short and severe disease processes to chronic undernutrition and vitamin deficiency,^52^^–^^54^ may explain the poor predictability of our selected covariates in explaining variation in HZ.

Further breaking down population variation in child anthropometric status/failure and hemoglobin/anemia into multiple administrative and political units suggests greater importance of local areas (communities) over districts or states. Prior multilevel analysis of poverty,^25^ catastrophic health spending,^55^ and undernutrition^56^ in India found the importance of micro-geographies that have been overlooked in policy discussions. The systematic component of variation ranged from 3.4–62.4% for states, 3.5–43.7% for districts, and 1.3–36.6% for communities, indicating a greater degree of clustering of covariates at larger geographic levels. It is also important to note that policies and programs designed with the exact same components may have differential impact on child undernutrition across states given the heterogeneity in the within-population variation and the proportion explained by same set of covariates.

The observed differences in population and individual variability in child undernutrition and the differing ability of a comprehensive set of proximate and distal covariates, jointly and individually, to explain these differences necessitate more targeted policy and practice interventions. In research concerning child nutrition, the inferential target should be more explicitly and clearly defined to be more informative as to where the majority of inequality exists and to what extent within- and between-population inequalities can be prevented and reduced. Given the persistently high burden of child undernutrition in India and other LMICs, efforts to improve the mean measures and underlying variability should occur in tandem, not separately.

## APPENDIX A. SUPPLEMENTARY DATA

eTable 1. Variance estimates and % variance partitioning in child undernutrition indicators using four-level random intercepts models, and % explained by a comprehensive set of risk factors, National Family Health Survey (NFHS-4; n = 139,116)

eTable 2. Parameter estimates for covariates included in adjusted four-level random intercepts model (model 2), National Family Health Survey (NFHS-4; n = 139,116)

eTable 3. Variance estimates and % variance partitioning in child undernutrition indicators using four-level random intercepts models, and % explained by a comprehensive set of risk factors from a secondary analysis with n = 25,605 children with additional data on paternal characteristics, National Family Health Survey (NFHS-4)

eTable 4. Variance estimates and % variance partitioning in binary indicators of child undernutrition using four-level random intercepts logistic models, and % explained by a comprehensive set of risk factors, National Family Health Survey (NFHS-4; n = 139,116)
