# Supplementary material for: Explaining Within- vs Between-Population Variation in Child Anthropometry and Hemoglobin Measures in India: A Multilevel Analysis of the National Family Health Survey 2015–2016
Source: J Epidemiol. 2020 Nov 5;30(11):485–96. doi: 10.2188/jea.JE20190064 (PMC7557174; doi:10.2188/jea.JE20190064)
Supplement: Supplementary file 1 [file je-30-485-s001.pdf]

eTable 1. Variance estimates and % variance partitioning in child undernutrition indicators using four-level random intercepts models, and % explained by a comprehensive set of risk factors, National Family Health Survey (NFHS-4; n=139,116)

	**Model 1**		**Model 2**		**% Explained**
	Variance Estimate (95% CI)	Variance Partitioning Coefficient (%)	Variance Estimate (95% CI)	Variance Partitioning Coefficient (%)	Model 2 vs. Model 1
**Height-for-age z-scorez-scores**					
State	0.06 (0.04, 0.10)	2.4%	0.02 (0.01, 0.04)	0.9%	62.4%
District	0.05 (0.05, 0.06)	2.1%	0.03 (0.02, 0.04)	1.3%	43.7%
Cluster	0.26 (0.25, 0.27)	10.1%	0.20 (0.18, 0.21)	8.4%	25.4%
Individual	2.21 (2.19, 2.22)	85.4%	2.09 (2.08, 2.11)	89.4%	5.4%
**Weight-for-age z-scorez-scores**					
State	0.09 (0.06, 0.15)	6.6%	0.05 (0.03, 0.09)	4.3%	43.2%
District	0.04 (0.04, 0.05)	3.0%	0.03 (0.02, 0.03)	2.1%	39.6%
Cluster	0.13 (0.13, 0.14)	9.3%	0.08 (0.08, 0.09)	6.8%	36.6%
Individual	1.17 (1.16, 1.18)	81.1%	1.08, 1.07, 1.09)	86.8%	7.5%
**Weight-for-height z-scorez-scores**					
State	0.08 (0.05, 0.14)	4.5%	0.06 (0.04, 0.11)	3.6%	22.0%
District	0.05 (0.05, 0.06)	2.9%	0.05 (0.04, 0.05)	2.7%	9.4%
Cluster	0.15 (0.14, 0.16)	8.3%	0.14 (0.13, 0.15)	7.9%	8.9%
Individual	1.55 (1.54, 1.57)	84.3%	1.52 (1.51, 1.54)	85.8%	2.0%
**Standardized Hemoglobin**					
State	0.04 (0.02, 0.07)	3.7%	0.04 (0.02, 0.06)	3.6%	3.4%
District	0.03 (0.03, 0.04)	3.3%	0.03 (0.03, 0.04)	3.2%	3.5%
Cluster	0.13 (0.13, 0.14)	12.9%	0.13 (0.12, 0.14)	12.8%	1.3%
Individual	0.83 (0.82, 0.84)	80.1%	0.83 (0.82, 0.84)	80.4%	0.2%

CI, confidence interval.

Model 1: age and sex.

Model 2: Model 1 + birth order, residence (urban/rural), household wealth, mother's height, mother's BMI, mother's age at marriage, child's dietary diversity, breastfeeding, drinking water availability, sanitation, safe stool disposal, infectious disease, household air quality, iodized salt, vitamin A supplementation, full vaccination, family planning, skilled birth attendant, ANC visits, child diarrhea in past 2 weeks, mother sought care for child with cough/fever.

% Explained refers to the change in variance estimates from Model 1 to Model 2 and is calculated as $(\frac{Model\ 1 - Model\ 2}{Model\ 1})$ x 100%.

eTable 2. Parameter estimates for covariates included in adjusted four-level random intercepts model (model 2), National Family Health Survey (NFHS-4; n=139,116)

	**HAZ**	**WAZ**	**WHZ**	**HZ**
Covariates	Parameter Estimate (SE)	Parameter Estimate (SE)	Parameter Estimate (SE)	Parameter Estimate (SE)
(Intercept)	-2.02 (0.05)	-2.03 (0.05)	-1.41 (0.06)	0.24 (0.04)
Age (48–59 months)	-0.87 (0.02)	-0.41 (0.01)	-0.03 (0.01)	-0.35 (0.01)
Age (36–47 months)	-0.91 (0.02)	-0.39 (0.01)	0 (0.01)	-0.23 (0.01)
Age (24–35 months)	-0.83 (0.02)	-0.31 (0.01)	-0.01 (0.01)	-0.15 (0.01)
Age (12–23 months)	-0.78 (0.01)	-0.2 (0.01)	0.05 (0.01)	0.12 (0.01)
Age (6–11 months)	(reference)	(reference)	(reference)	(reference)
Sex (male)	-0.09 (0.01)	-0.03 (0.01)	-0.05 (0.01)	0.02 (0.01)
Sex (female)	(reference)	(reference)	(reference)	(reference)
Birth order (6th or higher)	-0.18 (0.02)	-0.13 (0.02)	-0.03 (0.02)	0.06 (0.01)
Birth order (4th or 5th)	-0.13 (0.01)	-0.1 (0.01)	-0.04 (0.01)	0.05 (0.01)
Birth order (2nd or 3rd)	-0.09 (0.01)	-0.07 (0.01)	-0.03 (0.01)	0.03 (0.01)
Birth order (1st)	(reference)	(reference)	(reference)	(reference)
Residence (urban)	0.01 (0.01)	0.04 (0.01)	0.04 (0.01)	-0.03 (0.01)
Residence (rural)	(reference)	(reference)	(reference)	(reference)
Wealth (5)	0.4 (0.02)	0.37 (0.02)	0.2 (0.02)	-0.09 (0.02)
Wealth (4)	0.26 (0.02)	0.25 (0.01)	0.13 (0.02)	-0.05 (0.01)
Wealth (3)	0.2 (0.02)	0.18 (0.01)	0.09 (0.01)	-0.04 (0.01)
Wealth (2)	0.08 (0.01)	0.09 (0.01)	0.07 (0.01)	-0.02 (0.01)
Wealth (1)	(reference)	(reference)	(reference)	(reference)
Education (college)	0.31 (0.02)	0.26 (0.01)	0.11 (0.02)	-0.13 (0.01)
Education (higher)	0.22 (0.02)	0.15 (0.01)	0.03 (0.01)	-0.09 (0.01)
Education (secondary)	0.12 (0.01)	0.08 (0.01)	0.02 (0.01)	-0.06 (0.01)
Education (primary)	0.04 (0.01)	0.04 (0.01)	0.02 (0.01)	-0.03 (0.01)
Education (none)	(reference)	(reference)	(reference)	(reference)
Mother height (160+ cm)	1.11 (0.02)	0.79 (0.01)	0.22 (0.02)	-0.03 (0.01)
Mother height (155–159.9 cm)	0.83 (0.02)	0.59 (0.01)	0.16 (0.01)	-0.03 (0.01)
Mother’s height (150–154.9 cm)	0.59 (0.01)	0.43 (0.01)	0.12 (0.01)	-0.03 (0.01)
Mother’s height (145–149.9 cm)	0.34 (0.01)	0.24 (0.01)	0.06 (0.01)	-0.02 (0.01)
Mother’s height (<145 cm)	(reference)	(reference)	(reference)	(reference)
Mother’s BMI (2)	0.33 (0.01)	0.54 (0.01)	0.5 (0.01)	-0.04 (0.01)
Mother’s BMI (1)	0.21 (0.01)	0.29 (0.01)	0.26 (0.01)	-0.04 (0.01)
Mother’s BMI (0)	(reference)	(reference)	(reference)	(reference)
Mother married as a child (yes)	-0.01 (0.01)	0.00 (0.01)	0.02 (0.01)	0.00 (0.01)
Mother married as a child (no)	(reference)	(reference)	(reference)	(reference)

Dietary diversity (5)	0.01 (0.02)	0.04 (0.01)	0.05 (0.01)	-0.01 (0.01)
Dietary diversity (4)	0.03 (0.02)	0.08 (0.02)	0.07 (0.02)	0.02 (0.01)
Dietary diversity (3)	-0.02 (0.02)	0.03 (0.01)	0.05 (0.02)	0.04 (0.01)
Dietary diversity (2)	-0.02 (0.01)	0 (0.01)	0.01 (0.01)	0.01 (0.01)
Dietary diversity (1)	(reference)	(reference)	(reference)	(reference)
Breastfed (yes)	0.02 (0.01)	0.00 (0.01)	-0.01 (0.01)	-0.01 (0.01)
Breastfed (no)	(reference)	(reference)	(reference)	(reference)
Water (yes)	-0.04 (0.01)	-0.04 (0.01)	-0.01 (0.01)	0.01 (0.01)
Water (no)	(reference)	(reference)	(reference)	(reference)
Sanitation (yes)	0.04 (0.01)	0.02 (0.01)	0.00 (0.01)	-0.03 (0.01)
Sanitation (no)	(reference)	(reference)	(reference)	(reference)
Safe stool disposal (yes)	0.08 (0.01)	0.06 (0.01)	0.02 (0.01)	-0.01 (0.01)
Safe stool disposal (no)	(reference)	(reference)	(reference)	(reference)
Infectious disease (yes)	-0.01 (0.01)	-0.04 (0.01)	-0.05 (0.01)	0.04 (0.01)
Infectious disease (no)	(reference)	(reference)	(reference)	(reference)
Air quality (solid fuels, non-separate kitchen)	-0.05 (0.02)	-0.04 (0.01)	-0.01 (0.01)	0.00 (0.01)
Air quality (solid fuels, separate kitchen)	-0.01 (0.01)	-0.03 (0.01)	-0.03 (0.01)	-0.01 (0.01)
Air quality (no solid fuels)	(reference)	(reference)	(reference)	(reference)
Iodized salt (yes)	0.00 (0.02)	0.00 (0.01)	0.00 (0.02)	0.01 (0.01)
Iodized salt (no)	(reference)	(reference)	(reference)	(reference)
Vitamin A supplementation (yes)	0.00 (0.01)	-0.01 (0.01)	-0.01 (0.01)	0.00 (0.01)
Vitamin A supplementation (no)	(reference)	(reference)	(reference)	(reference)
Full vaccination (yes)	-0.05 (0.01)	-0.02 (0.01)	0.00 (0.01)	0.00 (0.01)
Full vaccination (no)	(reference)	(reference)	(reference)	(reference)
Family planning needs (unmet)	0 (0.01)	0.01 (0.01)	0.01 (0.01)	-0.01 (0.01)
Family planning needs (met)	(reference)	(reference)	(reference)	(reference)
Skilled birth attendant (yes)	0.04 (0.01)	0.04 (0.01)	0.03 (0.01)	-0.01 (0.01)
Skilled birth attendant (no)	(reference)	(reference)	(reference)	(reference)
Antenatal care visits (2)	-0.06 (0.01)	0.05 (0.01)	0.13 (0.01)	0.02 (0.01)
Antenatal care visits (1)	0.03 (0.01)	0.05 (0.01)	0.06 (0.01)	-0.02 (0.01)
Antenatal care visits (0)	(reference)	(reference)	(reference)	(reference)
Diarrhea (yes)	-0.05 (0.02)	-0.07 (0.01)	-0.04 (0.02)	0.01 (0.01)
Diarrhea (no)	(reference)	(reference)	(reference)	(reference)
Cough (yes)	0.00 (0.02)	-0.06 (0.01)	-0.05 (0.02)	-0.02 (0.01)
Cough (no)	(reference)	(reference)	(reference)	(reference)

BMI, body mass index; HAZ, height-for-age z-score; HZ, hemoglobin z-score; SE, standard error; WAZ, weight-for-age z-score; WHZ, weight-for-height z-score.
Model 1: age and sex.
Model 2: Model 1 + birth order, residence (urban/rural), household wealth, mother's height, mother's BMI, mother's age at marriage, child's dietary diversity, breastfeeding, drinking water availability, sanitation, safe stool disposal, infectious disease, household air quality, iodized salt, vitamin A supplementation, full vaccination, family planning, skilled birth attendant, ANC visits, child diarrhea in past 2 weeks, mother sought care for child with cough/fever.

eTable 3. Variance estimates and % variance partitioning in child undernutrition indicators using four-level random intercepts models, and % explained by a comprehensive set of risk factors from a secondary analysis with n=25,605 children with additional data on paternal characteristics, National Family Health Survey (NFHS-4)

	**Model 1**		**Model 2**		**Model 3**		**% Explained**	
	Variance Estimate (95% CI)	Variance Partitioning Coefficient (%)	Variance Estimate (95% CI)	Variance Partitioning Coefficient (%)	Variance Estimate (95% CI)	Variance Partitioning Coefficient (%)	Model 2 vs. Model 1	Model 3 vs. Model 2
**Height-for-age z-scorez-scores**								
State	0.06 (0.03, 0.11)	2.4%	0.03 (0.01, 0.05)	1.1%	0.03 (0.02, 0.06)	1.3%	57.32%	-13.01%
District	0.04 (0.03, 0.06)	1.7%	0.03 (0.02, 0.05)	1.2%	0.03 (0.02, 0.05)	1.2%	35.07%	-3.27%
Cluster	0.67 (0.63, 0.72)	25.9%	0.56 (0.52, 0.60)	23.8%	0.56 (0.52, 0.60)	23.8%	17%	0.81%
Individual	1.82 (1.78, 1.86)	70.0%	1.73 (1.70, 1.80)	73.8%	1.72 (1.69, 1.75)	73.7%	4.78%	0.77%
**Weight-for-age z-scorez-scores**								
State	0.10 (0.06, 0.17)	6.9%	0.06 (0.03, 0.10)	4.8%	0.06 (0.04, 0.11)	5.0%	40.34%	-3.07%
District	0.04 (0.03, 0.05)	2.8%	0.03 (0.02, 0.04)	2.0%	0.03 (0.02, 0.04)	2.0%	37.19%	1.21%
Cluster	0.35 (0.33, 0.38)	24.0%	0.27 (0.25, 0.29)	21.2%	0.26 (0.24, 0.28)	20.9%	24.35%	2.16%
Individual	0.97 (0.95, 0.99)	66.4%	0.90 (0.89, 0.92)	72.0%	0.89 (0.88, 0.91)	72.1%	6.89%	1.09%
**Weight-for-height z-scorez-scores**								
State	0.08 (0.05, 0.15)	4.6%	0.06 (0.04, 0.12)	3.6%	0.06 (0.03, 0.11)	3.6%	24.33%	1.8%
District	0.05 (0.04, 0.07)	2.8%	0.05 (0.03, 0.06)	2.6%	0.05 (0.03, 0.06)	2.6%	12.98%	1.09%
Cluster	0.45 (0.42, 0.48)	24.1%	0.42 (0.39, 0.45)	23.6%	0.41 (0.39, 0.45)	23.6%	6.46%	0.74%
Individual	1.27 (1.24, 1.29)	68.5%	1.24 (1.22, 1.27)	70.2%	1.24 (1.21, 1.26)	70.3%	2.08%	0.29%
**Standardized Hemoglobin**								
State	0.03 (0.02, 0.05)	2.8%	0.03 (0.02, 0.05)	2.6%	0.03 (0.02, 0.05)	2.6%	8.52%	-0.77%
District	0.02 (0.01, 0.03)	1.4%	0.01 (0.01, 0.03)	1.3%	0.01 (0.01, 0.03)	1.3%	9.38%	3.9%
Cluster	0.41 (0.39, 0.43)	38.4%	0.41 (0.38, 0.43)	38.4%	0.41 (0.38, 0.43)	38.4%	0.61%	0.11%
Individual	0.62 (0.60, 0.63)	57.4%	0.61 (0.60, 0.62)	57.7%	0.61 (0.60, 0.62)	57.7%	0.42%	0.07%

CI, confidence interval.
Model 1: age and sex.
Model 2: Model 1 + birth order, residence (urban/rural), household wealth, mother's height, mother's BMI, mother's age at marriage, child's dietary diversity, breastfeeding, drinking water availability, sanitation, safe stool disposal, infectious disease, household air quality, iodized salt, vitamin A supplementation, full vaccination, family planning, skilled birth attendant, ANC visits, child diarrhea in past 2 weeks, mother sought care for child with cough/fever.
Model 3: Model 2 + father's height, father's BMI, father's education, father's age.
% Explained refers to the change in variance estimates from Model 2 to Model 3 and is calculated as $(\frac{Model\ 2 - Model\ 3}{Model\ 2})$ x 100%.

eTable 4. Variance estimates and % variance partitioning in binary indicators of child undernutrition using four-level random intercepts logistic models, and % explained by a comprehensive set of risk factors, National Family Health Survey (NFHS-4; n=139,116)

	**Model 1**		**Model 2**		**% Explained**
	Variance Estimate (95% CI)	Variance Partitioning Coefficient (%)	Variance Estimate (95% CI)	Variance Partitioning Coefficient (%)	Model 2 vs. Model 1
**Stunting**					
State	0.26 (0.16, 0.42)	6.58%	0.16 (0.10, 0.27)	4.38%	37.1%
District	0.11 (0.09, 0.13)	2.77%	0.07 (0.06, 0.08)	1.90%	35.3%
Cluster	0.27 (0.25, 0.30)	6.90%	0.18 (0.16, 0.21)	4.96%	32.1%
Individual		88.75%		88.76%	
**Underweight**					
State	0.11 (0.07, 0.19)	2.95%	0.04 (0.02, 0.07)	1.18%	62.2%
District	0.09 (0.07, 0.10)	2.25%	0.04 (0.04, 0.05)	1.20%	49.7%
Cluster	0.32 (0.29, 0.34)	8.34%	0.22 (0.20, 0.25)	6.23%	29.4%
Individual		86.46%		91.40%	
**Wasting**					
State	0.16 (0.09, 0.27)	4.05%	0.14 (0.08, 0.24)	3.53%	14.0%
District	0.16 (0.14, 0.19)	4.08%	0.15 (0.13, 0.18)	3.87%	6.3%
Cluster	0.36 (0.33, 0.39)	8.99%	0.34 (0.31, 0.38)	8.73%	4.1%
Individual		82.88%		83.87%	

CI, confidence interval.
Model 1: age and sex.
Model 2: Model 1 + birth order, residence (urban/rural), maternal risk factors (household wealth, mother's height, mother's BMI, mother's age at marriage, child's dietary diversity, breastfeeding, drinking water availability, sanitation, safe stool disposal, infectious disease, household air quality, iodized salt, vitamin A supplementation, full vaccination, family planning, skilled birth attendant, ANC visits, child diarrhea in past 2 weeks, mother sought care for child with cough/fever).
VPC = 3.29 + cluster + district + state.
% Explained refers to the change in variance estimates from Model 1 to Model 2 and is calculated as $\left(\frac{Model\ 1 - Model\ 2}{Model\ 1}\right) \times 100\%$.
